# Effects of NaCl Treatment on Root Nodule Formation, Isoflavone Secretion in Soybean, and Nodulation Gene Expression in Rhizobia

**DOI:** 10.1264/jsme2.ME24023

**Published:** 2024-12-20

**Authors:** Yoshikazu Nitawaki, Takaaki Yasukochi, Shinya Naono, Akihiro Yamamoto, Yuichi Saeki

**Affiliations:** 1 Interdisciplinary Graduate School of Agriculture and Engineering, University of Miyazaki, Miyazaki 889–2192, Japan; 2 Graduate School of Agriculture, University of Miyazaki, Miyazaki 889–2192, Japan; 3 Faculty of Agriculture, University of Miyazaki, Miyazaki 889–2192, Japan

**Keywords:** saline soil, soybean, nodulation, isoflavones, *nodC* gene expression

## Abstract

We herein investigated the effects of salt (NaCl) stress on soybean nodulation by rhizobial strains. We specifically exami­ned: (1) the effects of NaCl on nodule maturity and positioning by inoculating three rhizobial strains (*Bradyrhizobium diazoefficiens* USDA110^T^, *Bradyrhizobium elkanii* USDA31, and *Sinorhizobium fredii* USDA191) onto soybean variety CNS, (2) the effects of the NaCl treatment on isoflavones (daidzein and genistein) secretion by CNS, (3) the effects of the NaCl treatment on gene expression induced by daidzein and genistein in rhizobia, and (4) the effects of the NaCl treatment on rhizobial growth. The results obtained were as follows: (1) the NaCl treatment delayed nodule development and reduced nodulation on the primary root following the USDA110^T^ inoculation, minimal sensitivity regarding nodule formation in the USDA 31 inoculation, and significantly increased the mature nodule number and nodules on the primary root following the USDA 191 inoculation. (2) The NaCl treatment significantly reduced the secretion of daidzein from soybean roots, but did not significantly affect that of genistein. (3) NaCl treatment induced a significant decrease in genistein-induced *nodC* expression in USDA110^T^, but not in USDA31, and also caused a significant reduction in daidzein-induced *nodC* expression, but not genistein-induced expression, in USDA191. (4) NaCl treatment reduced survivability under acidic conditions, but increased survivability under saline-alkaline conditions for USDA191 than bradyrhizobia. These results indicate that saline conditions give *S. fredii* a competitive advantage over *Bradyrhizobium* during soybean infection.

Saline soils are prevalent across various regions worldwide, including Central Asia, India, Pakistan, China, Australia, the United States, and Europe (Ivushkin *et al.*, 2019). They are caused by the accumulation of high salt concentrations in the surface layers due to the inadequate management of irrigation water, drought, seawater intrusion, and the abandonment of land after irrigation (Singh, 2015; Eswar *et al.*, 2019; Laiskhanov *et al.*, 2023). Saline soils are categorized into saline, sodic, and saline-sodic soils based on the type and quantity of salts present in the soil (Qadir *et al.*, 2014; Zaman *et al.*, 2018). Soil salinization is a global concern affecting more than 800 million ha of arable land worldwide and has a significant impact on crop production (FAO, 2008; Munns and Tester, 2008). Regions in which soil salinity and drought affect plant growth are expanding, covering 41–45% of the world’s land area (Gamalero *et al.*, 2020). High salt concentrations affect plant pH, leaf structure and function, osmotic pressure and turgor, the transpiration rate, and ion compositions in leaves, which reduce crop productivity (Khan *et al.*, 2014; Gamalero *et al.*, 2020). The‍ ‍global spread of saline soils poses substantial threats to crop productivity and endangers livelihoods worldwide. Additionally, the global population is projected to increase from approximately 8 billion in 2022 to around 9.7 billion by 2050 (United Nations, 2019). Therefore, research that focuses on enhancing crop productivity in saline soils is imperative.

Soybeans form symbiotic relationships with rhizobia. They obtain fixed nitrogen from and provide carbohydrates to symbiotic rhizobia. During soybean root initiation, signaling substances, such as the isoflavones daidzein and genistein, are produced and secreted into the rhizosphere (Matsuda *et al.*, 2023). Rhizobia in the soil receive these substances and synthesize lipo-chitooligosaccharides known as Nod factors (Wulandari *et al.*, 2022). The basic structures of Nod factors are synthesized sequentially by various enzyme groups, namely, NodA, NodB, and NodC, which are encoded by the nodulation genes *nodA*, *nodB*, and *nodC*, respectively (Downie, 2014). Their specific substitutions, which affect host specificities, differ depending on the rhizobial strains (D’Haeze and Holsters, 2002). Soybean roots recognize Nod factors, leading to the expression of genes involved in rhizobial infection and the formation of root nodules (Janczarek *et al.*, 2015). This process of rhizobial infection is affected by various soil environmental factors, including moisture, temperature, pH, nitrogen compounds, and the type and concentration of salts (Nishida and Suzaki, 2018; Ferguson *et al.*, 2019; Nakei *et al.*, 2022). Dardanelli *et al.* (2010) previously reported that a salt treatment inhibited the secretion of isoflavones, such as daidzein, and the intermediate naringenin from the roots of soybean variety Osumi. A number of studies have exami­ned of *nod* gene expression in soybean rhizobia and the recognition of Nod factors by soybeans in relation to environmental factors (Duzan *et al.*, 2004; Shiro *et al.*, 2016). However, ana­lyses that combine isoflavone secretion from soybeans and *nod* gene expression under salt stress conditions have yet to be performed. Additionally, high nodulation rates of *Ensifer* (*Sinorhizobium*) *fredii* have been detected in alkaline soils in Okinawa and Vietnam as well as in salt-accumulated soils in China and Brazil (Saeki *et al.*, 2005; Suzuki *et al.*, 2008; Han *et al.*, 2009; Li *et al.*, 2011). Some studies have evaluated salt tolerance in rhizobia using neutral media with 0–4% NaCl (Mukherjee and Samaddar, 1997; Djedidi *et al.*, 2011). However, an evaluation of salt tolerance in relation to pH has not yet been conducted. We previously investigated the effects of NaCl on the nodulation of soybean variety CNS by various rhizobial strains and showed distinct variations in nodulation rates that were dependent on the rhizobial strain inoculated under NaCl treatments (Nitawaki *et al.*, 2021). We demonstrated that nodule numbers were not affected by NaCl treatments following inoculations with *B. diazoefficiens* USDA110^T^ and *B. elkanii* USDA31, whereas they significantly increased under 20–50‍ ‍mM NaCl following an inoculation with *S. fredii* USDA191. Moreover, USDA191 exhibited higher competitiveness in a mixed inoculation under NaCl treatments. However, the mechanisms responsible for superior nodulation by *S. fredii* under saline conditions remain unclear.

Therefore, the present study investigated the mechanisms by which *S. fredii* achieved superior nodulation efficiency over *Bradyrhizobium* spp. soybean rhizobia under salt (NaCl) stress conditions. We initially exami­ned the effects of NaCl on root nodule formation following inoculations with three types of rhizobia under NaCl treatment conditions. We then investigated changes in the secretion of isoflavones from the roots. We supplemented rhizobia with two types of isoflavones and assessed the effects of NaCl on *nodC* gene expression in rhizobia. We also exami­ned the salt tolerance of rhizobia on media. Based on the results obtained, we investigated the effects of NaCl on the infection process leading to the establishment of the symbiotic relationship between soybean variety CNS and various rhizobial strains and the dominance of *S. fredii* in saline-alkaline soils.

## Materials and Methods

### Soybean and rhizobium

In this study, the soybean variety CNS (*Glycine max* [L.] Merr.) was employed as the plant material, and the rhizobial strains *B. diazoefficiens* USDA110^T^, *B. elkanii* USDA31, and *S. fredii* USDA191, all compatible with variety CNS for nodulation, were utilized (Nitawaki *et al.*, 2021). We herein refer to the latter species as *S. fredii* instead of *Ensifer* (*Sinorhizobium*) *fredii*, following the common convention of using the genus name with the suffix “rhizobium” for rhizobial species. All strains were cultured on Yeast-extract Mannitol Agar (YMA, Vincent, 1970) medium and stored at 4°C until used.

### Effects of the NaCl treatment on nodule development

Experiments were conducted to evaluate the effects of the NaCl treatment on nodule development in soybeans inoculated with rhizobia. Rhizobia were transferred from YMA culture plates to Yeast-extract Mannitol Broth (YMB, Vincent, 1970) and cultured at 28°C in the dark with continuous agitation at 120‍ ‍rpm for 3 days for *S. fredii* and 5 days for *Bradyrhizobium* species until a stationary phase of 10^8^‍ ‍cells‍ ‍mL^–1^ was reached. Cultures were then diluted with sterilized water to 10^6^‍ ‍cells‍ ‍mL^–1^ to prepare inoculants (Nitawaki *et al.*, 2021). The upper pots of Leonard jars (volume of 900‍ ‍mL) were used as cultivation pots, filled with vermiculite, and supplemented with a 1/4 strength N-free hydroponic solution containing 0‍ ‍mM (control) or 50‍ ‍mM NaCl (Saeki *et al.*, 2000; Nitawaki *et al.*, 2021). The same hydroponic solution was added to the lower part of the jar, and the jars were covered with aluminum foil and autoclaved at 121°C for 20‍ ‍min for sterilization. Soybean seeds were surface-sterilized with 70% ethanol and sodium hypochlorite (0.25% available chlorine) before sowing (Nitawaki *et al.*, 2021). Sterilized seeds were individually sown and then covered with vermiculite, with each seed receiving 1‍ ‍mL of an inoculant. Soybean cultivation took place in a plant growth chamber for 21 days, with a 16-h light period at 28°C and an 8-h dark period at 23°C. The number and size of nodules on the roots were exami­ned on days 12, 15, 18, and 21 post-seed inoculation. Nodules with a diameter ≥1‍ ‍mm were classified as “mature nodules”, whereas those <1‍ ‍mm were classified as “immature nodules”, with nodule numbers being measured accordingly. The experiment was replicated 3–5 times, and a statistical ana­lysis comparing different NaCl concentrations was performed using Welch’s *t*-test.

### Effects of NaCl on the formation of nodules on primary and lateral roots

To assess the infection rate based on the nodulation site, experiments were conducted independently of mature and immature nodule counts. Seeds were sown separately in 0‍ ‍mM (control) and 50‍ ‍mM NaCl treatments using the same medium as described above. Three strains of rhizobia were simultaneously inoculated using the same method as described above. Soybean cultivation followed the same procedure as described above, in a plant growth chamber for 21 days with a 16-h light period at 28°C and an 8-h dark period at 23°C. Nodule counts on both the primary and lateral roots were recorded for each treatment on days 12, 15, 18, and 21 after the soybean seed inoculation. The experiment was replicated 5–9 times, and a statistical ana­lysis comparing nodulation counts on the primary and lateral roots between the different NaCl concentrations was performed using Welch’s *t*-test.

### Effects of NaCl on soybean isoflavone secretion

To prepare soybean seedlings, approximately 50‍ ‍mL of vermiculite was added to a 50-mL conical tube, followed by 20‍ ‍mL of distilled water. The tube was covered with aluminum foil and autoclaved at 121°C for 20‍ ‍min. Sterilized soybean seeds were individually sown, and the surface was covered with vermiculite. Soybean cultivation was conducted in a plant growth chamber under the conditions of 16‍ ‍h of light at 28°C and 8‍ ‍h of darkness at 23°C for 5–7 days after sowing. Seedlings were then collected from the tubes and vermiculite was carefully removed in pure water. The three soybean seedlings were submerged in a 50-mL conical tube containing 40–45‍ ‍mL of 1/4 strength N-free hydroponic solution containing 0 or 50‍ ‍mM NaCl and cultivated under the same conditions in the plant growth chamber for 48 h. After cultivation, the hydroponic solution was collected for isoflavone measurements. Isoflavones were extracted following the method described by Sugiyama *et al.* (2016). Briefly, the hydroponic solution was adjusted to pH 3.0 with hydrochloric acid, filtered through a 0.45-μm DISMIC filter (Advantec), supplemented with 6-methoxyflavone as the internal standard, and then made up to 100‍ ‍mL. The solution was then passed through a Sep-Pak C18 Plus short cartridge (Waters), followed by elution with methanol to recover isoflavones. Isoflavones were concentrated under nitrogen gas, dried, and re-dissolved in methanol. Isoflavones were quantitatively analyzed using HPLC (LC20AD; Shimadzu) with CAPCELL PAK C18 MGII S5 4.6×250‍ ‍mm (SHISEIDO) and UV detection at wavelengths of 254 and 260‍ ‍nm. The column temperature was maintained at 40°C and the flow rate was set to 1.0‍ ‍mL‍ ‍min^–1^. The methanol concentration gradient was programmed as follows: 50–60% from 0 to 20‍ ‍min, 60–80% from 20 to 40‍ ‍min, 80–95% from 40 to 41‍ ‍min, 95% from 41 to 43‍ ‍min, and 95–50% from 43 to 55‍ ‍min. The peak areas of isoflavones were normalized using the peak area ratio of the internal standard, and isoflavone contents were measured. Dry weight measurements of the soybean roots utilized for isoflavone secretion were also performed. Isoflavone secretion was evaluated per unit dry weight and per individual plant. The experiment was replicated 5 times, and a statistical ana­lysis of isoflavone levels between different NaCl concentrations was performed using Welch’s *t*-test.

### Effects of NaCl on *nodC* gene expression in rhizobia

To examine the effects of NaCl on *nodC* gene expression, three strains of rhizobia (*B. diazoefficiens* USDA110^T^, *B. elkanii* USDA31, and *S. fredii* USDA191) were used. NaCl concentrations were adjusted to 0, 30, and 100‍ ‍mM in the medium for the ana­lysis of *nodC* expression (Shiro *et al.*, 2016; Nitawaki *et al.*, 2021). In the *nodC* gene expression ana­lysis in rhizobia, we used the housekeeping genes *sigA* and *gyrB* as reference genes (Beck *et al.*, 1997; Rocha *et al.*, 2015). Following the method by Shiro *et al.* (2016), the three rhizobial strains derived from YMA plates were inoculated in HEPES-MES (HM) liquid medium containing arabinose (Cole and Elkan, 1973; Sameshima *et al.*, 2003) and then cultured in the dark at 28°C with continuous agitation at 120‍ ‍rpm. *S. fredii* was cultured for 3 days, while *Bradyrhizobium* strains were cultured for 5 days until reaching a stationary phase with a concentration of 10^8^‍ ‍cells‍ ‍mL^–1^. According to Shiro *et al.* (2016), a 50-mL aliquot of the culture was mixed with 50‍ ‍mL of fresh HM liquid medium containing daidzein or genistein at a concentration of 10‍ ‍μM and NaCl at concentrations of 0, 60, and 200‍ ‍mM (final concentration of isoflavones: 5‍ ‍μM, NaCl: 0, 30, and 100‍ ‍mM). Cultures were incubated with shaking at 100‍ ‍rpm at 28°C under dark conditions for 24 h. Following the incubation, the bacterial suspension was centrifuged at 4°C and 9,000‍ ‍rpm for 5‍ ‍min using a refrigerated centrifuge to collect the rhizobial precipitate, followed by RNA extraction. RNA extraction was performed using ISOGEN (Nippon Gene) after the addition of 1‍ ‍μL of a recombinant RNase inhibitor (Takara Bio) and drying RNA in a vacuum concentrator, followed by dissolution in 20‍ ‍μL of RNase-free dH_2_O. Genomic DNA was eliminated using Recombinant DNaseI (Takara Bio) according to the protocol, and cDNA was synthesized using PrimeScript RT (Takara Bio) following the established protocol. Real-time PCR (denaturation at 95°C for 5‍ ‍s and extension at 63°C for 30 s; 50 cycles) was performed using the Thermal Cycler Dice Real-Time System TP800 (Takara Bio) with specific primers for each gene (Table 1) and Luna Universal qPCR Master Mix (New England Biolabs). The specificity of PCR products was confirmed by denaturation at 95°C for 15‍ ‍s and annealing at 60–95°C (Lang *et al.*, 2008). The experiment was replicated 5 times. The relative expression levels of the *nodC* gene in the isoflavone-treated group were compared to those in the untreated group for each NaCl treatment using Dunnett’s test (Pfaffl, 2001; Livak and Schmittgen, 2001). Furthermore, Dunnett’s test was performed to assess the significance of differences between each NaCl treatment and the 0‍ ‍mM NaCl control group. This approach enabled us to evaluate the effects of NaCl stress on *nodC* gene expression induced by two types of isoflavones.

### The NaCl tolerance of rhizobia on YMA medium

To evaluate the salt tolerance of rhizobia, the colony area on YMA plate medium with adjusted pH and NaCl concentrations was exami­ned (Mukherjee and Samaddar, 1997; Djedidi *et al.*, 2011). The pH of the medium (pH 5.0, 6.8, and 9.0, adjusted with 0.5 M HCl and 0.5 M NaOH) and NaCl concentrations (0.01, 0.1, 0.3, 0.5, 0.7, and 0.9%) were adjusted in YMA medium. After culturing on YMB medium, bacterial density was measured under a microscope and adjusted to 10^6^‍ ‍cells‍ ‍mL^–1^ with sterile water. Thereafter, 0.7‍ ‍μL of the adjusted bacterial suspension was spotted onto YMA medium and cultured in the dark at 28°C. After an incubation for 240 h, colony areas were measured using ImageJ (https://imagej.net/ij/) and the relative area ratios of rhizobial colonies were evaluated against the colony area on YMA medium containing 0.01% NaCl as the control. Cultures were triplicates for pH 6.8 and six replicates for pH 5 and 9, and a statistical ana­lysis was performed using Dunnett’s test.

### Statistical ana­lysis

Statistical ana­lyses were conducted using R v.4.1.1 (R Core Team: https://www.R-project.org/).

## Results

### Effects of the NaCl treatment on nodule development

**Fig. 1.**
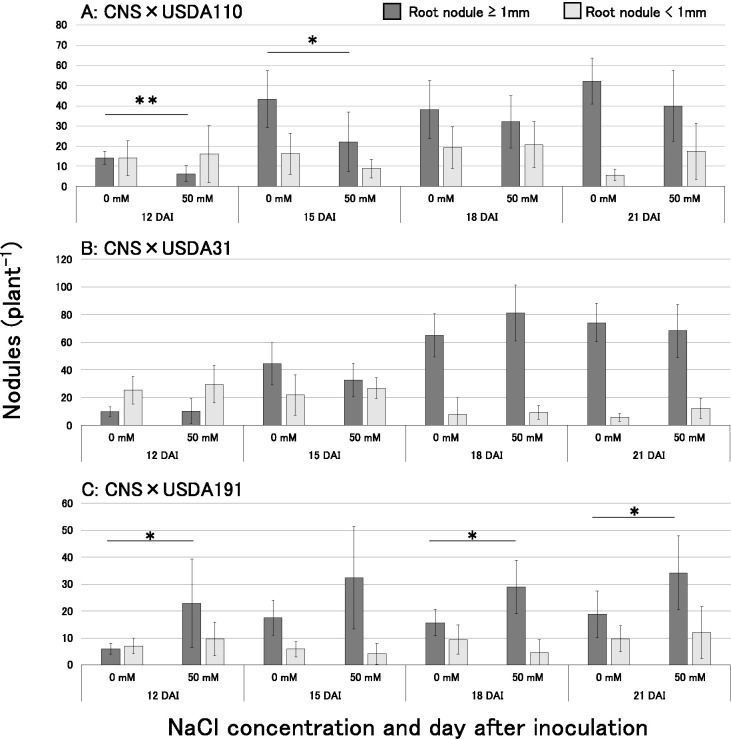
Mature and immature root nodule numbers on soybean hosts inoculated with rhizobia under saline conditions: (A) *Bradyrhizobium diazoefficiens* USDA110^T^, (B) *Bradyrhizobium elkanii* USDA31, and (C) *Sinorhizobium fredii* USDA191. Data are shown as the mean±S.D. (*n*=3–5), with asterisks indicating significant differences in Welch’s *t*-test (*: *P*<0.05, **: *P*<0.01).

The present study investigated the effects of salt (NaCl) on the nodulation process mediated by the three rhizobial strains using soybean variety CNS. Fig. 1A, B, and C show the results obtained on nodule numbers. Following the USDA110^T^ inoculation (Fig. 1A), mature nodule numbers were significantly lower 12 and 15 days after inoculation (DAI) under the 50‍ ‍mM NaCl treatment than under the control treatment (0‍ ‍mM NaCl). However, no significant difference was observed by 18 and 21 DAI, with almost equal nodule numbers being counted. Conversely, the number of immature nodules under the 50‍ ‍mM NaCl treatment was similar to that under the control treatment. Following the USDA31 inoculation (Fig. 1B), the numbers of mature and immature nodules at each time point post-rhizobial inoculation under the 50‍ ‍mM NaCl treatment were similar to those under the control treatment. Following the USDA191 inoculation (Fig. 1C), the number of mature nodules was significantly higher at 12, 18, and 21 DAI under the 50‍ ‍mM NaCl treatment than under the control treatment. However, no significant differences were observed in the number of immature nodules at any DAI between both treatments.

### Effects of NaCl on the formation of nodules on primary and lateral roots

**Fig. 2.**
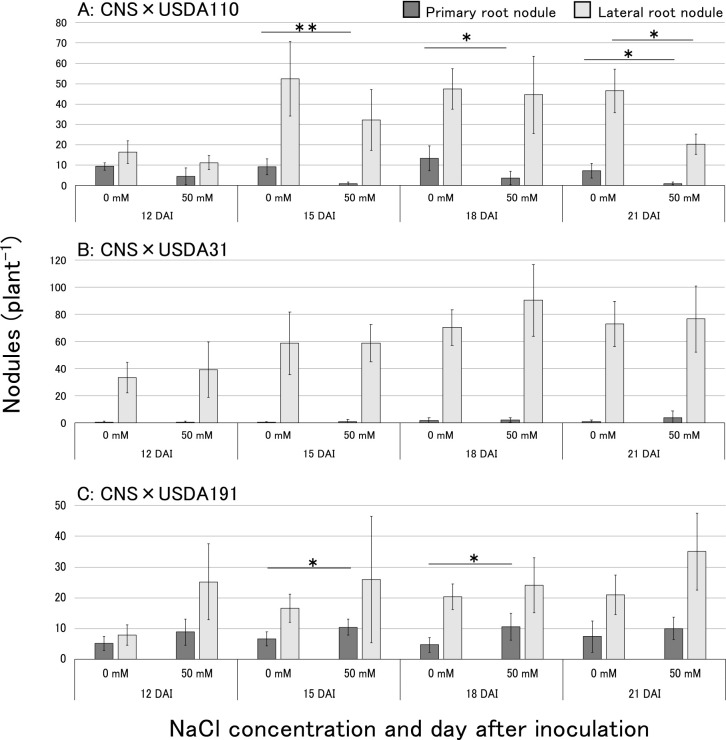
Nodule numbers on primary or lateral roots of host soybeans inoculated with rhizobia under saline conditions: (A) *Bradyrhizobium diazoefficiens* USDA110^T^, (B) *Bradyrhizobium elkanii* USDA31, and (C) *Sinorhizobium fredii* USDA191. Data are shown as the mean±S.D. (*n*=5–7), with asterisks indicating significant differences in Welch’s *t*-test (*: *P*<0.05, **: *P*<0.01).

Fig. 2 shows the results of nodule formation on the primary and lateral roots on 12–21 DAI. Based on the results shown in Fig. 2, following the USDA110^T^ inoculation, the number of nodules on primary roots was significantly lower on 15, 18, and 21 DAI under the 50‍ ‍mM NaCl treatment than under the control treatment (Fig. 2A). However, following the USDA31 inoculation, the number of nodules on the primary roots was similar under both treatments (Fig. 2B). Following the USDA191 inoculation, the number of nodules on the primary roots on 15 and 18 DAI was significantly higher under the 50‍ ‍mM NaCl treatment (Fig. 2C). Nodule numbers on the lateral roots also slightly increased under the 50‍ ‍mM NaCl treatment.

### Effects of NaCl on soybean isoflavone secretion

**Fig. 3.**
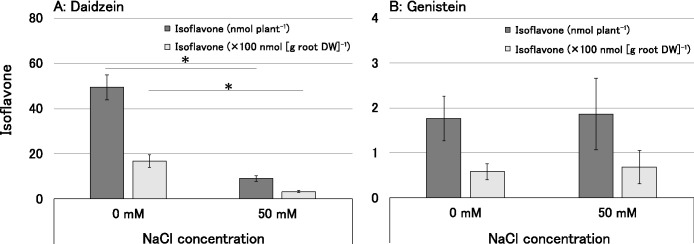
Effects of NaCl treatments on isoflavone secretion from soybean roots: (A) daidzein, and (B) genistein. Data are shown as the mean±S.D. (*n*=3–5), with asterisks between NaCl treatments indicating significant differences in Welch’s *t*-test (*: *P*<0.05).

Fig. 3 shows the results obtained on the concentrations of isoflavones secreted from the roots of CNS in the absence of rhizobial inoculations. A comparison of the amounts of two isoflavones, daidzein and genistein, using 5-day-old seedlings showed low levels of secretion (0.66–1.17‍ ‍nmol daidzein plant^–1^ and 0.20–0.22‍ ‍nmol genistein plant^–1^), with no significant differences under the control and NaCl treatments (data not shown). However, in the experiment using 7-day-old seedlings, the secretion of daidzein increased under the control treatment, whereas a significant decrease in daidzein secretion was observed under the NaCl treatment (Fig. 3A). In contrast, the secretion of genistein was lower than that of daidzein, with levels ranging from 58–68‍ ‍nmol (g root DW)^–1^, and no significant difference was observed due to the NaCl treatment (Fig. 3B). Despite a 28-fold difference between daidzein and genistein secretion under the control treatment, the decrease in daidzein secretion under to the NaCl treatment resulted in a difference in concentrations of less than 5-fold.

### Effects of NaCl on *nodC* gene expression in rhizobia

**Fig. 4.**
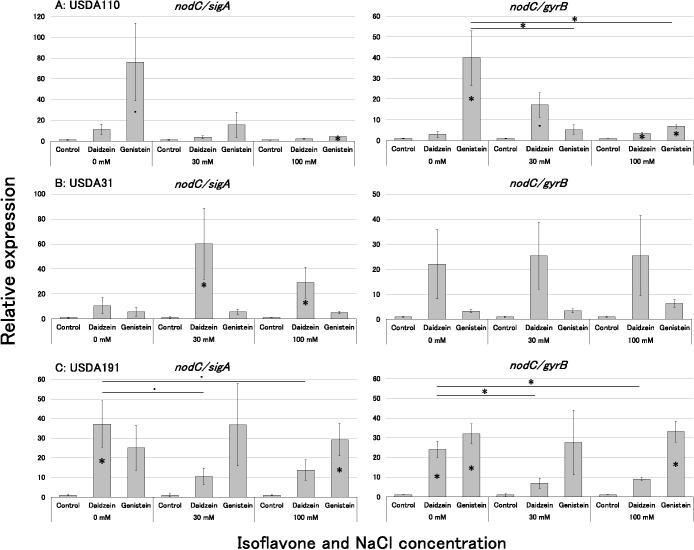
Relative expression of the *nodC* gene in soybean rhizobia at each NaCl concentration. The values represent the expression levels of the *nodC gene* in the presence of an isoflavone (5‍ ‍μM) relative to those of the control at each NaCl concentration in the medium. Data are shown as the mean±S.E. (*n*=5). Expression levels were normalized to the housekeeping genes *sigA* and *gyrB*. In multiple comparisons, Dunnett’s tests were conducted using the absence of an isoflavone at each NaCl concentration as the control and between NaCl concentrations using 0‍ ‍mM NaCl as the control for relative expression levels. ・ and * in the bar indicate a significant difference from the control, and ・ and * between bars indicate a significant difference from the control (・: *P*<0.1, *: *P*<0.05).

Changes in *nodC* expression under various NaCl concentrations were exami­ned. Similar expression levels were obtained in both correction results, despite large differences in variability (Fig. 4). In *B. diazoefficiens* USDA110^T^, the significant up-regulation of *nodC* expression was observed in the *nodC*/*gyrB* expression under the control with genistein treatment. *nodC*/*sigA* expression increased with the genistein treatment, similar to *nodC/gyrB* expression (Fig. 4A). However, a significant decrease was observed in *nodC/gyrB* expression with the genistein treatment as NaCl concentrations increased. Although no significant difference was noted, a similar decrease was observed in *nodC*/*sigA* expression (Fig. 4A). In contrast, relative expression levels in the daidzein treatment zone increased regardless of the NaCl concentration; however, no significant changes were noted in expression levels under the different NaCl treatments, similar to the genistein treatment. In *B. elkanii* USDA31, the NaCl treatments had no significant effect on expression levels (Fig. 4B). *nodC* gene expression in response to the isoflavone treatment showed no significant difference in the control. However, *nodC* expression with the daidzein treatment was significantly higher under the 30‍ ‍mM and 100‍ ‍mM NaCl treatments than under the control treatment in *nodC/sigA* expression. Although there was no significant difference, *nodC/gyrB* expression with the daidzein treatment was similar to *nodC/sigA* expression. In *S. fredii* USDA191, *nodC/gyrB* expression significantly‍ ‍in­creased with both the genistein and daidzein treatments, and similar changes were detected in *nodC/sigA* expression (Fig. 4C). However, *nodC* expression with the daidzein treatment significantly decreased with increases in the concentration of NaCl in both *sigA* and *gyrB* corrections. On the other hand, no significant decrease in expression was observed in either correction zone with the genistein treatment. The results obtained from the *nodC* expression ana­lysis of rhizobia indicated similar outcomes in both corrections based on the *sigA* and *gyrB* genes, showing different reactions to varying NaCl concentrations depending on the strain of bacteria and the type of isoflavone.

### The NaCl tolerance of rhizobia on YMA medium

**Fig. 5.**
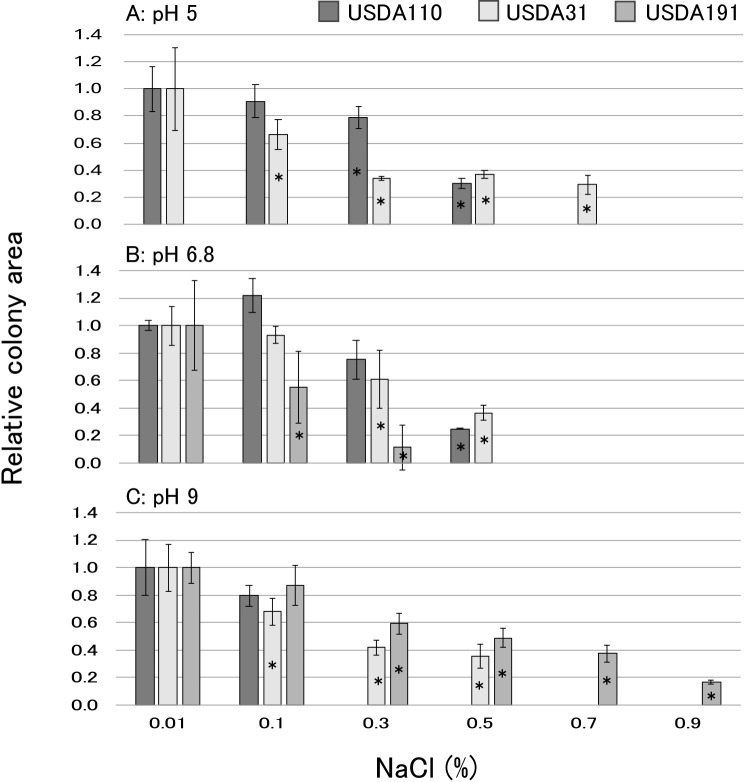
Salt tolerance of rhizobia on YMA medium. Colony areas after an incubation for 240‍ ‍h were evaluated against the colony area on YMA medium containing 0.01% NaCl as the control. Data are shown as the mean±S.D. (*n*=3–6). * in the bar indicates a significant difference from the control (Dunnett’s test, *: *P*<0.05).

The results obtained on the NaCl tolerance of rhizobia on YMA medium are shown in Fig. 5. Colony growth by all strains was inhibited under the NaCl treatments; however, NaCl tolerance varied among the strains. *B. diazoefficiens* USDA110 showed tolerance up to 0.5% NaCl at pH 5–6.7, but did not grow at 0.3% NaCl and pH 9 (Fig. 5A). *B. elkanii* USDA31 exhibited broad tolerance, showing resistance up to 0.7% NaCl at pH 5 and up to 0.5% NaCl at pH 6.8–9 (Fig. 5B). *S. fredii* USDA191 displayed different characteristics from bradyrhizobia, showing no growth at pH 5. It exhibited tolerance up to 0.3% NaCl at pH 6.8 and up to 0.9% NaCl at pH 9 (Fig. 5C).

## Discussion

We herein exami­ned the effects of a NaCl treatment on the nodulation process of the soybean variety CNS by three rhizobial strains (*B. diazoefficiens* USDA110^T^, *B. elkanii* USDA31, and *S. fredii* USDA191). Regarding bradyrhizobia, mature nodule formation by USDA110^T^ showed an approximately 3-day delay under the 50‍ ‍mM NaCl treatment (Fig. 1A), while the NaCl treatment did not affect nodulation by USDA31 (Fig. 1B). Conversely, the NaCl treatment facilitated mature nodule formation by USDA191 (Fig. 1C). Our previous study on nodulation dominance by these three rhizobial strains in mixed inoculation experiments revealed differences in nodulation dominance under NaCl treatments (Nitawaki *et al.*, 2021). Specifically, the nodulation occupancy of strain USDA191 significantly increased at higher NaCl concentrations, whereas that of USDA110^T^ significantly decreased. Other studies noted the prevalent nodulation of *Sinorhizobium* spp. in alkaline soils in Okinawa and Vietnam as well as in saline-accumulated soils in China and Brazil (Saeki *et al.*, 2005; Suzuki *et al.*, 2008; Han *et al.*, 2009; Li *et al.*, 2011). The present results suggest that the high nodulation rate of *S. fredii* under saline conditions contributes to its dominance of nodulation in saline-alkaline soils. Tu (1981) and Duzan *et al.* (2004) reported that NaCl stress reduced the responsiveness of soybean root hairs to rhizobial Nod factors. Therefore, in the USDA110^T^ inoculation, the recognition of a USDA110^T^-synthesized Nod factor by CNS root hairs may have been reduced under NaCl treatment conditions, which may have delayed nodulation. However, in the case of the USDA191 inoculation, the responsiveness of soybean root hairs to rhizobial Nod factors increased under the NaCl treatment, resulting in distinct effects of NaCl on soybean root hair recognition of Nod factor between the USDA191 and USDA110^T^ inoculations. Consequently, the effects of NaCl on soybean root hair recognition of Nod factor appeared to be less pronounced than those on the rhizobial recognition of soybean isoflavones. Although this issue warrants further study, it may be due to differences in the structures of Nod factors produced by rhizobia. Strain USDA110^T^ synthesizes a single-structure Nod factor, whereas USDA191 produces multiple Nod factors (D’Haeze and Holsters, 2002). There may be advantages to synthesizing multiple Nod factors for nodulation under saline conditions.

We noted a decrease in daidzein secretion under the 50‍ ‍mM NaCl treatment (Fig. 3A). This result was similar to the findings of Dardanelli *et al.* (2010) showing that a NaCl treatment inhibited the secretion of daidzein and the intermediate naringenin from soybean roots. Therefore, the reduced secretion of daidzein from CNS roots may be due to the effects of NaCl on the generation and subsequent secretion of isoflavones. Conversely, although genistein secretion levels were lower than those of daidzein, no discernible effect of NaCl on genistein secretion was observed (Fig. 3B). USDA110^T^ exhibited high responsiveness to genistein, leading to a significant increase in *nodC* expression under the control treatment. However, responsiveness to daidzein was lower than that to genistein under the control treatment. The response to genistein decreased under the NaCl treatments (Fig. 4A), leading to the significant inhibition of *nodC* gene expression. This result suggested that the synthesis of Nod factors was suppressed, thereby delaying infection of the host soybean (Fig. 1A and 2A). Regarding USDA31, although the significant expression of *nodC/sigA* under the daidzein treatment was not observed with the control treatment, *nodC* gene expression was generally efficient regardless of the NaCl treatment (Fig. 4B). Additionally, the response to genistein was weaker than that to daidzein; however, the increase in *nodC* gene expression remained unchanged with the NaCl treatment. Therefore, the delay in rhizobial infection due to the NaCl treatment was not significant (Fig. 1B and 2B). Following the USDA31 inoculation, more nodules formed on the lateral roots than on the primary roots regardless of the presence of NaCl (Fig. 2B). In a previous study (Nitawaki *et al.*, 2021), when a mixed inoculation of these three strains (USDA31, USDA110^T^, and USDA191) was performed, the nodulation occupancy of USDA31 was markedly lower than those of the other two strains. These results indicate that infection by USDA31 was not affected by NaCl; however, its infectivity was lower than that by the two other strains under the culture conditions employed. The response of strain USDA191 to daidzein was significantly weaker than that of other rhizobia as the NaCl concentration increased, while its response to genistein remained unchanged (Fig. 4C). Therefore, despite the decrease in daidzein secretion from soybean roots, the expression of *nod* genes remained unaffected due to the stable secretion of genistein. In contrast to USDA110^T^, the sustained high expression of *nod* genes by genistein in USDA191 resulted in the maintenance of infectivity. Consequently, infection by USDA191, which maintained the expression of genistein-inducible *nod* genes under the NaCl treatment, appeared to be stronger than that by USDA110^T^, leading to an increase in nodulation occupancy. These results show the changes in isoflavone secretion from soybeans and the responses of rhizobia to genistein and daidzein under saline conditions, and offer insights into the mechanisms underlying the high nodulation occupancy of *S. fredii* in saline soils.

The results obtained on the NaCl tolerance of rhizobia on YMA medium under three pH conditions (Fig. 5) were attributed to *Bradyrhizobium* being alkali-producing bacteria and *Sinorhizobium* being acid-producing bacteria on YM medium (Suzuki *et al.*, 2008). Additionally, the present results indicate that the survivability of USDA191 was inferior under acidic conditions and superior under saline-alkaline conditions to *Bradyrhizobium* (Fig. 5). Since the pH range in the culture pots used for soybean cultivation was 6.8–7.0, and 50‍ ‍mM NaCl was approximately 0.3% NaCl, the salt tolerance of USDA191 on the medium does not serve as evidence for the superiority of USDA191 in nodule formation under saline conditions in this study. However, nodule formation by *S. fredii* was observed even under the culture conditions employed herein, indicating that its characteristics on medium differ from the actual rhizobial ecology in soil and the soybean rhizosphere. *S. fredii* has a high nodule occupancy rate in soybean cultivation under saline-alkaline conditions and is dominant in saline-alkaline soils (Saeki *et al.*, 2005; Suzuki *et al.*, 2008; Han *et al.*, 2009; Li *et al.*, 2011), suggesting that its salt tolerance on medium is one of the physiological characteristics that allow it to dominate in saline-alkaline soils.

In the present study, we focused on isoflavone concentrations in 7-day-old seedlings and analyzed *nodC* gene expression 24‍ ‍h after the isoflavone treatment. Therefore, the results of this study alone cannot fully explain the significant increases observed in the rate of nodule formation and the number of primary root nodules (Fig. 1C and 2C). In another study on different rhizobia, such as *Rhizobium tropici* CIAT899, the expression of nodulation genes was observed in response to a NaCl treatment even without *nod* gene inducers (Guasch-Vidal *et al.*, 2013). Therefore, *S. fredii* may up-regulate *nod* gene expression under saline conditions, even in the absence of isoflavones. More detailed studies, such as time-course ana­lyses of isoflavone secretion from roots and *nod* gene expression in response to salt or isoflavone treatments, are necessary to further explore this aspect. Although additional research is needed to understand the promotion of *S. fredii* nodulation under saline-alkaline conditions, the present study has provided insights into the mechanisms by which soybean infection by *S. fredii* is superior to *Bradyrhizobium* under saline conditions.

## Citation

Nitawaki, Y., Yasukochi, T., Naono, S., Yamamoto, A., and Saeki, Y. (2024) Effects of NaCl Treatment on Root Nodule Formation, Isoflavone Secretion in Soybean, and Nodulation Gene Expression in Rhizobia. *Microbes Environ ***39**: ME24023.

https://doi.org/10.1264/jsme2.ME24023

## Figures and Tables

**Table 1.** Primers for qRT-PCR targeting the *nodC* gene in rhizobia

Target gene	Forward (5′–3′)	Reverse (5′–3′)
*nodC* for *Bradyrhizobium diazoefficiens*	TGGACGGGATTGACGATTG	GTGTGGAGCGAGAAGCCG
*nodC* for *Bradyrhizobium elkanii*	TGGACGGTGCTGACGATTG	TGTGAAGCGAGAAGCCGAG
*nodC* for *Sinorhizobium fredii*	GTCGACGATCCTGATGATTGC	TGTGCAGCGAAAACCCAAG
*sigA*	GCATGTATCTGCGCGAGATG	TCGTCGCGCCAGATGA
*gyrB*	CTGCGCGGCAAGATCC	GGTGATCAGCGTGCCGA
